# Supplementary material for: Complexome profiling of the Chlamydomonas psb28 mutant reveals TEF5 as an early PSII assembly factor
Source: Plant Cell. 2025 Mar 17;37(6):koaf055. doi: 10.1093/plcell/koaf055 (PMC12164590; doi:10.1093/plcell/koaf055)
Supplement: koaf055_Supplementary_Data [file koaf055_supplementary_data.zip › psb28_interactive.zip!/plots/intensity/qualityPlots/peptides/rbcL_Cre-1.g2717040.t1.p1.html]

### Cre-1.g2717040.t1.p1

Trivial name: rbcL  
SeqCoverage [median]: 31.60  
Mass: 52.54 kDa  
  
 

| Sequence | MissedCleavage | Mass [Da] | wtIntensity | muIntensity | Score |
| --- | --- | --- | --- | --- | --- |
| ALRLEDLR | 1 | 984.57 | 0.00 | 9632.68 | 113.41 |
| ALRLEDLRIPPAYVK | 2 | 1753.03 | 0.00 | 1061.96 | 30.18 |
| AMHAVIDR | 0 | 911.46 | 3558.21 | 3694.65 | 101.64 |
| AMHAVIDRQR | 1 | 1195.62 | 0.00 | 6553.91 | 89.05 |
| AQAETGEVK | 0 | 931.46 | 22124.21 | 25808.84 | 131.50 |
| DDENVNSQPFMR | 0 | 1450.61 | 1176.64 | 1790.12 | 167.67 |
| DLAREGGDVIR | 1 | 1199.63 | 0.00 | 2935.16 | 62.80 |
| DNGLLLHIHR | 0 | 1186.66 | 573.79 | 805.68 | 70.10 |
| DTDILAAFR | 0 | 1020.52 | 41393.06 | 23344.09 | 206.50 |
| EGGDVIR | 0 | 744.38 | 8239.79 | 36546.47 | 96.51 |
| EVTLGFVDLMR | 0 | 1278.66 | 823.72 | 0.00 | 79.15 |
| FLFVAEAIYK | 0 | 1199.66 | 408.56 | 0.00 | 90.66 |
| GGLDFTK | 0 | 736.38 | 751.03 | 0.00 | 79.47 |
| GGLDFTKDDENVNSQPFMR | 1 | 2168.98 | 11422.04 | 20160.71 | 129.42 |
| IPPAYVK | 0 | 786.46 | 5646.94 | 8486.84 | 81.72 |
| LEDLRIPPAYVK | 1 | 1412.80 | 1633.54 | 6717.48 | 116.54 |
| LEGEREVTLGFVDLMR | 1 | 1862.96 | 0.00 | 2132.43 | 62.65 |
| LEGEREVTLGFVDLMRDDYVEKDR | 3 | 2883.41 | 0.00 | 12793.52 | 52.91 |
| LTYYTPDYVVR | 0 | 1388.70 | 32318.77 | 60807.59 | 223.32 |
| MSGGDHLHSGTVVGK | 0 | 1480.71 | 45357.39 | 31877.52 | 110.25 |
| NHGIHFR | 0 | 879.45 | 0.00 | 830.04 | 77.75 |
|  |  |  | 175427.7 | 255979.7 |  |
